# Identification of Internal Reference Genes in Peripheral Blood Mononuclear Cells of Cattle Populations Adapted to Hot Arid Normoxia and Cold Arid Hypoxia Environments

**DOI:** 10.3389/fgene.2021.730599

**Published:** 2022-02-01

**Authors:** Preeti Verma, Ankita Sharma, Monika Sodhi, Manish Tiwari, Prince Vivek, Ranjit S. Kataria, S. K. Nirajan, Vijay K. Bharti, Pawan Singh, S. S. Lathwal, Vishal Sharma, Nampher Masharing, Manishi Mukesh

**Affiliations:** ^1^ Animal Biotechnology Division, ICAR-National Bureau of Animal Genetic Resources, Karnal, India; ^2^ Animal Biotechnology Center, ICAR-National Dairy Research Institute, Karnal, India; ^3^ DRDO-Defense Institute of High-altitude Research, Leh, India; ^4^ Department of Livestock Production Management, ICAR-National Dairy Research Institute, Karnal, India

**Keywords:** reference genes, expression stability, qRT-PCR, normalization, hot arid, cold arid, cattle, PBMCs

## Abstract

To estimate gene expression in a reliable manner, quantitative real-time polymerase chain reaction data require normalisation using a panel of stably expressed reference genes (RGs). To date, information on an appropriate panel of RGs in cattle populations reared at cold arid high-altitude hypoxia and hot arid tropical normoxia environments is not available. Therefore, the present study was carried out to identify a panel of stably expressed RGs from 10 candidate genes (*GAPDH, RPL4, EEF1A1, RPS9, HPRT1, UXT, HMBS, B2M, RPS15,* and *ACTB*) in peripheral blood mononuclear cells (PBMCs) of cattle populations reared at cold arid high-altitude hypoxia and hot arid normoxia environments. Four different statistical algorithms: geNorm, NormFinder, BestKeeper, and RefFinder were used to assess the stability of these genes. A total of 30 blood samples were collected: six adult heifers each of Ladakhi (LAC) and Holstein Frisian crosses (HFX) and 4 Jersey (JYC) cows from cold arid high-altitude hypoxia environments (group I) and five adult heifers each of Sahiwal (SAC), Karan Fries (KFC), and Holstein Friesian (HFC) cows from hot arid normoxia environments (group II). Combined analysis of group I and group II resulted in identification of a panel of RGs like *RPS9, RPS15,* and *GAPDH* that could act as a useful resource to unravel the accurate transcriptional profile of PBMCs from diverse cattle populations adapted to distinct altitudes.

## Introduction

India has been blessed with several cattle breeds of *Bos indicus* lineage adapted to various agro-climatic zones from high land to hot tropical regions. Leh-Ladakh, also known as a “COLD DESERT” is a part of the western Himalayan agro-climatic and high-altitude temperate sub-agro-climatic zone in India. Ladakh is situated at an altitude of 3,500–5,500 m with difficult terrain and harsh climate conditions such as extreme temperature (−40°C in winter and 35°C in summer), low humidity (25–40%), low precipitation (80–300 mm), and low oxygen level (nearly 60–70% of the oxygen concentration at sea level). In spite of harsh weather, this region is blessed with several unique livestock populations such as yak, cattle, horses, sheep, goat, donkey, and double-hump camel. Over thousands of years of the evolutionary process, these animals have developed the special ability to survive in cold and hypoxia environments prevalent in Ladakh. Amongst all the livestock species, the native cattle of Ladakh known as “Ladakhi cattle” are the major livestock species that play an important role in the agriculture economy of the region. The cattle from the Trans-Himalayan region of Ladakh are short in stature and well-adapted to the high-altitude environment. Based on morphometric data on 275 animals and genetic characterization using microsatellite markers (unpublished data), this cattle population was observed to be highly distinct from native cattle breeds adapted to other agroclimatic zones of India. Recently, our group was successful in delineating the distinct transcriptome signatures of peripheral blood mononuclear cells (PBMCs) of high-altitude-adapted Ladakhi cattle and tropically adapted Sahiwal cattle (Verma et al., 2018a; Verma et al., 2018b). In the last few years, the purity of Ladakhi cattle is believed to have declined due to widespread intermixing with Jersey cattle. However, considering the unique hypoxia-tolerant characteristics of Ladakhi cattle, preserving its purity will be a key for long-term conservation and sustainable utilization.

On the other hand, India has also been blessed with a huge native cattle genetic resource base which has adapted to hot and tropical conditions. For example, native cattle breeds like Sahiwal, Tharparkar, Rathi, Gir, Ongole etc. are known for their superior thermotolerance as compared to their exotic counterparts of the *Bos taurus* lineage. The superior cellular tolerance ability of PBMCs from native cattle has been shown in a few studies published by our group (Kishore et al., 2013; Kishore et al., 2014; Sharma et al., 2019) using PBMCs as the cellular model. However, native cattle breeds are also facing genetic dilution due to crossbreeding with exotic germplasm in order to enhance milk production. These native cattle populations that are adapted to distinct altitudes might have acquired a distinct gene pool during the course of the evolutionary process. Such genetic resources with remarkable adaptive traits could be an interesting resource to mine gene expression and the mechanism underlying changes associated with adaptation to cold arid and hot arid environments. It would be interesting to define the importance of various genes in conferring adaptation to cattle populations adapted to diverse altitudes through targeted gene expression analysis. Quantitative real-time polymerase chain reaction (qRT-PCR) has been widely employed to quantify the expression of target genes of interest in different tissues/cells exposed to a variety of experimental conditions. In spite of many of its advantages, this tool is prone to analytical variations arising due to differing amounts of starting material, pipetting errors, and differing efficiencies of RNA extraction and reverse transcription (Vandesompele et al., 2002; Huggett et al., 2005; Bustin et al., 2009). To overcome the limitations of experimental variation, the use of appropriate internal control genes (ICGs) or reference genes (RGs) to successfully normalise the RT-qPCR data has been reported in several studies (Bustin 2010; Castigliego et al., 2010; Crookenden et al., 2017; Die et al., 2017; Sang et al., 2018). The approach to identify a suitable panel of RGs during various experimental/physiological conditions has also been reported in different livestock species (Aggarwal et al., 2013; Kapila et al., 2013; Zhu et al., 2015; Jatav et al., 2016; Li et al., 2016; Kaur et al., 2018). However, to the best of our knowledge, no comparative data on suitable RGs are available for cattle populations reared at cold arid high-altitude and hot arid tropical regions. The present study was planned to identify a panel of stably expressed RGs in PBMCs of six cattle populations from the high-altitude cold arid region of Leh-Ladakh and hot arid tropical climate of India.

## Materials and Methods

### Ethics Statement and Animal Selection

The blood sampling of animals was performed in accordance with the relevant guidelines and regulations as approved by the Institutional Animal Ethics Committee (IAEC) of ICAR-National Bureau of Animal Genetics Resources (NBAGR), Karnal. The study has included three cattle populations from the cold arid high-altitude region of Leh-Ladakh, *viz*., Ladakhi cattle (native), Jersey cattle (exotic), and Frieswal cattle {Sahiwal x Holstein Frisian cross}, and three cattle populations from the hot dry and semi-arid condition of Haryana state, *viz*., Sahiwal cattle (native), Holstein Frisians cattle (exotic), and Karan Fries cattle {Tharparkar x Holstein Frisian}. Ladakhi cattle (LAC) are the native breed of the *Bos indicus* lineage and are naturally adapted to the high-altitude region of Ladakh. Jersey cattle (JYC) are of the *Bos taurus* lineage and originated from temperate regions, while Frieswal cattle (HFX) are a cross-bred cattle population. Both Jersey and Frieswal cattle are non-native to Leh-Ladakh and have been reared in limited organized farms in the region since the last 2 decades. Amongst the cattle populations selected from the hot semi-arid region, Sahiwal cattle (SAC) are a very popular native cattle breed of *Bos indicus* lineage and are known for their adaptation potential to hot dry and tropical conditions. Holstein Frisian cattle (HFC) are of *Bos taurus* lineage and are non-native to India. However, Holstein Frisian cattle have been widely used in India in various cross-breeding programmes to enhance milk production of local breeds. Karan Fries cattle (KFC) are a popular cross-breed that were developed several decades ago by crossing Tharparkar cattle (native) with Holstein Frisian. Therefore the study has included two populations of native cattle: one from high-altitude (Ladakhi cattle; LAC) and the other from hot arid tropical regions (Sahiwal cattle; SAC); and two populations of cross-breeds and two populations of exotic cattle from two extreme altitudes. The geographical coordinates of the sampling site representing the hot arid climate were latitude– 29° 3′ 56.7828″ N, and longitude 76° 2′ 25.7892″ E. The geographical coordinates of the sampling site from the high-altitude region of Ladakh were latitude– 34° 9′ 9.3168″ N, and longitude 77° 34′ 37.3764″ E. About 7–8 ml of whole blood samples was collected aseptically from the external jugular vein of the animals in sterile EDTA-coated vacutainer tubes.

**FIGURE 1:**
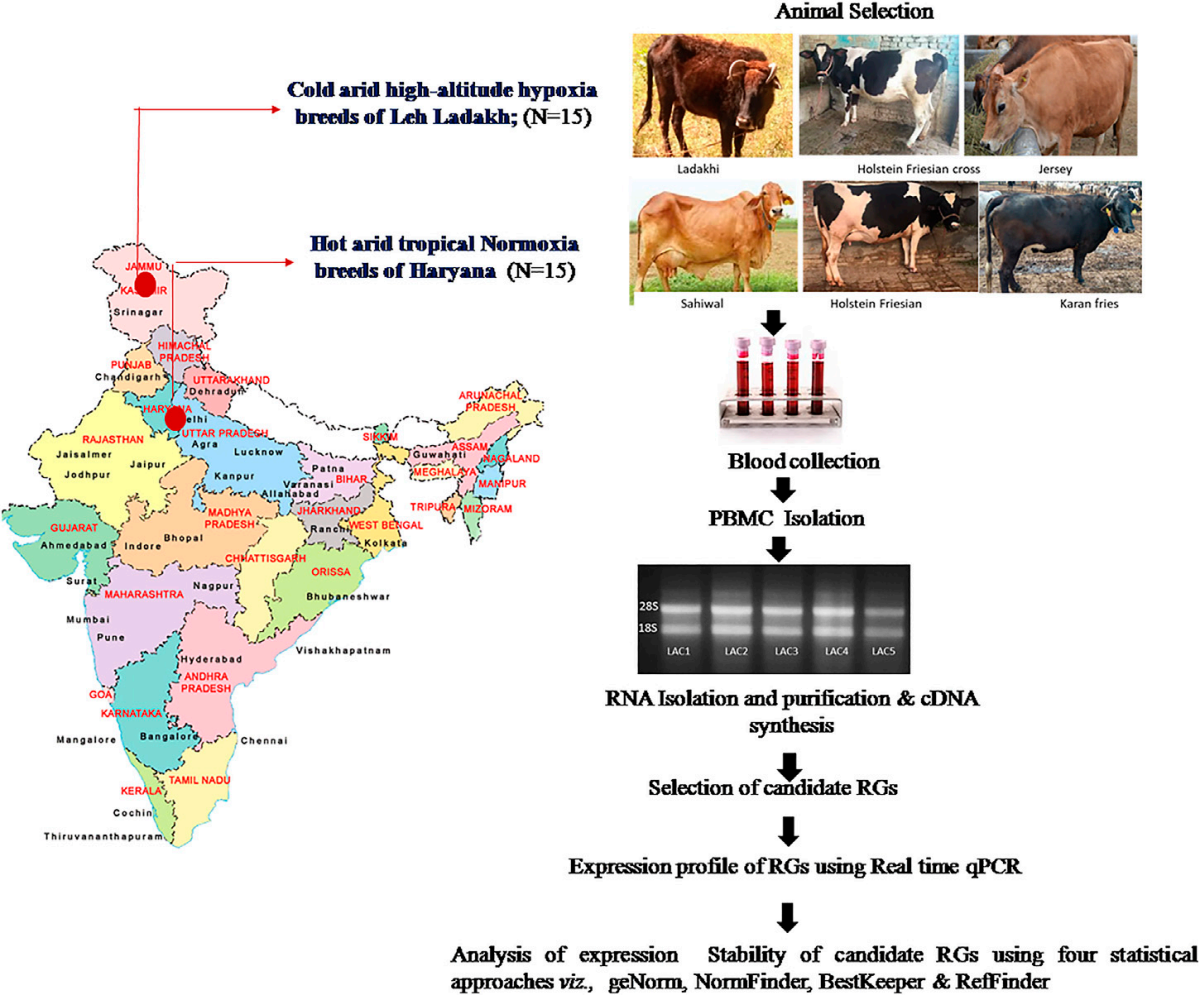
The entire workflow of the present study conducted using PBMCs of cattle breeds from cold arid high-altitude hypoxia and hot arid normoxia groups. The map of India in Figure 1 was created with the help of Smartdraw software- https://www.smartdraw.com)*.*

In total, 30 blood samples were collected from adult heifers; five each of Sahiwal (SAC), Karan Fries (KFC), and Holstein Friesian (HFC) cows from the hot arid normoxia environment and 5 heifers each of Ladakhi (LAC), Holstein Frisian crosses (HFX), and Jersey (JYC) cows from the cold arid high-altitude hypoxia environment. The entire workflow of the qPCR experiment is depicted in Figure 1.

### Peripheral Blood Mononuclear Cells Isolation, RNA Extraction, and cDNA Synthesis

Immediately after collection, the blood samples were processed to isolate peripheral blood mononuclear cells (PBMCs) using the density gradient centrifugation method as described by Verma et al. (2018b). In brief, blood was diluted in a 1:1 ratio with 1 × PBS (Ca^2+^ and Mg^2+^ free; Hyclone, Utah) and was gently over laid on a Histopaque-1077 (Sigma-Aldrich Inc., United States) followed by centrifugation at 4000 RPM for 30 min at RT. After removing the buffy coat in a separate 15 ml tube, cells were treated with 2 ml of chilled RBC lysis buffer for 5 min at RT and washed twice with 1 × PBS (Ca^2+^ and Mg^2+^ free; Hyclone, Utah). Finally, the cells were suspended in 1.0 ml of ice cold Trizol reagent (Invitrogen, Carlsbad, California), homogenized, and stored at −80°C. Total RNA was extracted using Trizol reagent according to the manufacturer’s instructions (Invitrogen, Corp., CA, United States). The extracted RNA was further purified using RNeasy mini kit columns (Qiagen, Germany). To remove the traces of genomic DNA, rnase free DNase enzyme was used according to the manufacturer’s instructions (Qiagen, Germany). RNA concentration and purity were checked using a NanoDrop ND-1000 spectrophotometer (Thermo Scientific, United States) and Experion Bio-analyzer (Bio-Rad, United States). The OD_260_/OD_280_ absorption ratio for different samples varied from 1.92 to 2.10. The RNA integrity number (RIN) values for all purified RNA samples were in the satisfactory range (6.5–8.5). The RNA integrity of each sample was also confirmed by visualizing 28S and 18S ribosomal bands on 1.5% agarose gel. The cDNA was synthesized using a Revertaid First strand cDNA synthesis kit (Fermentas, United States) as described in our previous studies (Kishore et al., 2013; Verma et al., 2018b). Briefly, first strand cDNA was synthesized with 200 ng of purified RNA, oligo-dT (18) primer, dNTP mix, random primers, RiboLockRNase inhibitor, and M-MuLV reverse transcriptase supplied with RevertAid First Strand cDNA Synthesis (Thermo Scientific, CA, United States), using the program: 25°C for 5 min, 50°C for 60 min, and 70°C for 15 min. Before using them as templates for qPCR, each of the cDNA samples was diluted 1:4 (v:v) with DNase/RNase free water. The quality of 30 cDNAs was confirmed by amplifying the *GAPDH* gene using a similar protocol to that described for qPCR except for the final dissociation protocol. A small aliquot of amplified products for all the samples was run on 2.5% agarose gel to check the primer specificity and amplification quality.

### Selection of Candidate Reference Genes and Real-Time Quantitative PCR Primers

In the present study, 10 candidate genes belonging to different functional groups were selected for evaluation as suitable RGs (Table 1). The candidate genes included in the study were; glyceraldehyde 3-phosphate (*GAPDH*), ribosomal protein L-4 (*RPL4*), eukaryotic elongation factor 1 alpha (*EEF1A1*), ribosomal protein S9 (*RPS9*), hypoxanthine guanine phosphoribosyl transferase 1 (*HPRT1*), ubiquitin expressed transcript (*UXT*), hydroxyl methylbilane (*HMBS*), beta 2-microglobulin (*B2M*), ribosomal protein S15 (*RPS15*), and beta actin (*ACTB*). The primers specific for these 10 RGs were available in the laboratory and have been utilized successfully in several of our previous studies (Kapila et al., 2013; Jatav et al., 2016; Kaur et al., 2018). The information about sequences, amplicon length, and annealing temperature for each primer pair are summarized in Table 1.

**TABLE 1:** Gene symbol, primer sequence, amplicon size, slope, PCR efficiency and (*R* ^2^) of RGs for each evaluated RG.

Gene Symbol	Accession no	Primer sequences	Annealing temp (°C)	Amplicon Size (bp)	Slope	PCR efficiency (%)	*R* ^2^
*ACTB*	NM_173979.3	F:5′GCGTGGCTACAGCTTCACC3′	60	56	−3.14	108.20	0.996
		R:3′TTGATGTCACGGACGATTTC5′					
*GAPDH*	NM_001034034.2	F:5′TGGAAAGGCCATCACCATCT3′	60	60	−3.58	90.20	0.827
		R:3′CCCACTTGATGTTGGCAG5′					
*EEF1A1*	NM_174535.2	F:5′CATCCCAGGCTGACTGTGC3′	60	101	−3.30	100.92	0.986
		R:3′TGTAAGCCAAAAGGGCATGC5′					
*B2M*	XM_002691119.4	F:5′CTGCTATGTGTATGGGTTCC3′	60	101	−3.27	102.20	0.998
		R:3′GGAGTGAACTCAGCGTG5′					
*HMBS*	NM_001046207.1	F:5′CTTTGGAGAGGAATGAAGTG3′	60	80	−3.20	105.21	0.996
		R:3′AATGGTGAAGCCAGGAGGA5′					
*RPL4*	NM_001014894.1	F:5′TTGGAAACATGTGTCGTGGG3′	60	101	−3.32	101.2	0.922
		R:3′GCAGATGGCGTATCGCTTCT5′					
*RPS15*	NM_001037443.2	F:5′GAATGGTGCGCATGAATGTC3′	60	101	−3.54	91.6	0.989
		R:3′GACTTTGGAGCACGGCCTAA5′					
*RPS9*	NM_001101152.2	F:5′CCTCGACCAAGAGCTGAAG3′	60	54	−3.34	99.25	0.941
		R:3′CCTCCAGACCTCACGTTTGTT5′					
*UXT*	NM_001037471.2	F:5′TGTGGCCCTTGGATATGGTT3′	60	101	−3.22	104.4	0.997
		R:3′GGTTGTCGCTGAGCTCTGTG5′					
*HPRT11*	NM_001034035.2	F:5′GAGAAGTCCGAGTTGAGTTT3′	60	101	−3.64	88.06	0.987
		R:3′GGCTCGTAGTGCAAATGAAG5′					
*HIF1A*	NM_174339.3	F:5′TGAAGGCACAGATGAATTGC3′	60	129	−3.16	103	0.991
		R:3′GTTCAAACTGAGTTAATCCC5′					
*EPAS1*	NM_174725.2	F:5′AGCAAGCCTTCCAAGACATGA3′ R:3′GCTTGTCCGGCATCAAAGAG5′	60	90	−3.10	114	0.995
*HSP70*	JN604432.1	F:5′AACATGAAGAGCGCCGTGGAGG 3′	60	171	−2.90	120	0.990
		R:5′GTTACACACCTGCTCCAGCTCC3′					
*HSP27*	NM_001014911.1	F: 5′TAC​ATT​TCC​CGT​TGC​TTC​A3′	60	78	−3.20	104	0.998
		R: 3′GGA​CAG​AGA​GGA​GGA​GAC5′					

qPCR, efficiencies for each primer calculated pair-wise from a six-point standard curve using a five-fold dilution series of pooled DNA of Ladakhi and Sahiwal cow PBMCs

*R* ^2:^ correlation coefficient of the slope of the standard curve.

### Real-Time Quantitative PCR Reference Genes Transcripts

Quantitative PCR was performed in a 10 μL reaction volume containing 4 μl of diluted cDNA and 6 μl of master mix composed of 5 μL of 2X LightCycler 480 SYBR Green (Roche Life Science, Germany), 0.4 μL each of 10 μM forward and reverse primers, and 0.2 μL of DNase/RNase free water. Each qRT-PCR reaction was performed in duplicate to check the quality by assessing intra-assay variation. The amplification was carried out in a 96-well block using a LightCycler 480-II real-time PCR instrument (Roche Life Science, Germany) with the following conditions; 2 min at 50°C, 10 min at 95°C, 40 cycles of 15 s at 95°C (denaturation), and 1 min at 60°C (annealing + extension). In order to evaluate the quality of qPCR reactions in terms of nonspecific amplification and primer-dimer formation, a dissociation curve for each gene was obtained by increasing the temperature from 60°C to 95°C. A six-point relative standard curve was prepared for each gene by using five-fold serial dilutions of pooled cDNA samples in duplicate. The amplification specificity for each primer was checked by the presence of a single band of expected size on 2.5% agarose gel (Supplementary Figure S1), and also by observing the single melt curve peak after completion of qPCR (Supplementary Figure S2). The qPCR data for each gene were extracted using the “second derivative maximum” method (Rasmussen 2001) as computed by Light Cycler software 3.5 for subsequent analysis.

### Analysis of Expression Stability of Candidate Reference Genes

The qPCR data recorded for each gene were subsequently analysed to evaluate the expression stability. Four statistical approaches, *viz*., geNorm (Vandesompele et al., 2002), NormFinder (Andersen et al., 2004), BestKeeper (Xu et al.,. 2016), and the RefFinder web tool (Xie et al., 2012) were used to calculate the expression stability and ranking of RGs in the high-altitude hypoxia cold arid group (LAC, JYC, and HFX) and normoxia hot arid group (SAC, KFC, and HFC). The C_q_ values of each RG were exported to an Excel work sheet and modified as per the requirement of the software. Like for creating an input file for geNorm and NormFinder analysis, the C_q_ values were first transformed into relative quantities by using formula 2−^∆CT^, in which ∆C_q_ = corresponding C_q_ value - minimum C_q_ value. geNorm calculated the expression stability of individual genes on the basis of the M value which indicates the stability in expression of a gene. The genes with smaller M values (<1.5) are considered to have higher expression stability (Xu et al., 2016). In addition, geNorm was also used to conduct pair-wise variation analysis (Vn/Vn+1) in order to select the optimal number of RGs to normalise the target gene expression data. The cut off value of Vn/Vn+1 < 0.15 was used to decide the optimal number of RGs to be employed for calculating the normalisation factor (Vandesompele et al., 2002). This analysis is based on the principal that the expression ratio of the two best RGs will always remain similar across samples. The NormFinder software calculated the stability values of each RG based on inter- and intra-group variations. Similar to geNorm, for Normfinder analysis, the C_T_ values were first transformed into relative quantities. However, for Bestkeeper analysis, C_T_ values were not transformed into relative quantities. The BestKeeper algorithm was used to calculate gene expression variation based on cycle threshold values (C_q_), crossing point standard deviation [{SD (±CP)} <1], and coefficient of variance (CV [%CP]). In BestKeeper analysis, genes with low SD (<1), low CV, and high coefficient of correlation (r) are generally considered stably expressed and vice versa.

Finally, the RefFinder tool (https://www.heartcure.com.au/reffinder/) was also employed to estimate the overall ranking of the 10 RGs by assigning an appropriate weight to each gene (Xie et al., 2012). The RefFinder analysis integrates the outcome of geNorm, NormFinder, and BestKeeper tools to provide an overall final ranking of RGs.

## Results

### Primer Specificity, Amplification Efficiency, and Descriptive Statistics

This study evaluated the expression stability of 10 RGs (*GAPDH*, *RPL4, EEF1A1, RPS9, HPRT1, UXT, HMBS, B2M, RPS15*, and *ACTB*) in PBMCs of cattle types from cold arid high-altitude hypoxia and hot arid normoxia environments. The specificity of each primer pair was ascertained by the presence of the single specific amplicon of expected size on 2.5% agarose gel (Supplementary Figure S1). The melt curve plot of each RG showed a single peak suggesting the highly specific nature of primer pairs used (Supplementary Figure S2). The amplification efficiencies estimated from the six-point standard curve (generated from five-fold serial dilution of pooled cDNA) ranged from 88 to 110%. The slope values of the standard curve for different RGs ranged between −3.05 and −3.5 which was within the acceptable limit (−2.96 to −3.6). Based on the overall evaluation of the melt curve, amplification efficiencies, and slope values, it could be safely assumed that RT-qPCR data for each primer pair were of high quality (Table 1).

**FIGURE 2:**
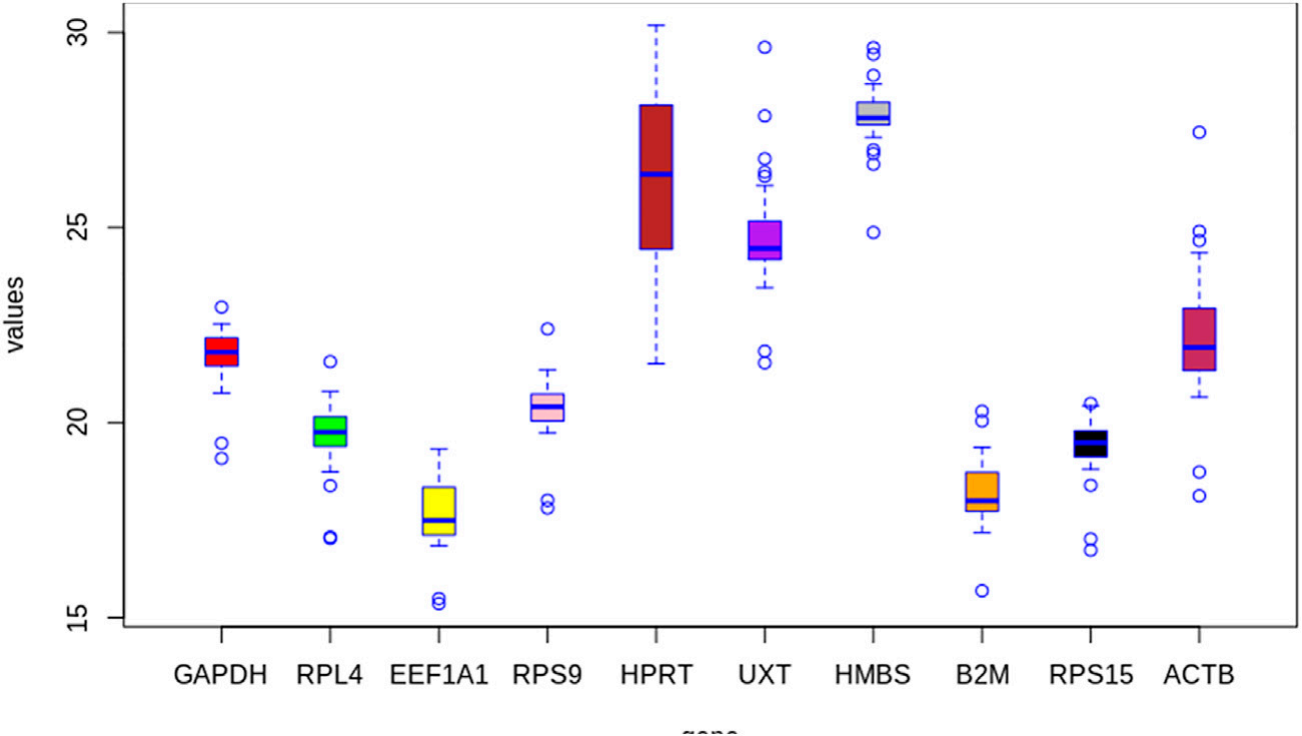
Box whisker plot showing expression levels of individual candidate RGs in the combined group (cold arid and hot arid). The data are presented as quantification cycle (CP) values of each gene in the box-whisker diagram. The median is shown as a line across the box while whiskers indicate maximum and minimum values.

The average raw Cq values of individual RGs across all PBMCs in cold arid and hot arid groups (combined analysis) are summarized in Table 2. The average Cq values of individual RGs were quite variable and ranged from 17.66 (*EEF1A1*) to 27.86 (*HMBS*). On the basis of their average C_t_ scores, the 10 RGs were classified into group-I (abundantly expressed), group-II (moderately expressed), and group-III (least expressed). Group-1 included *EEF1A1*, *B2M*, *RPS15,* and *RPL4* genes with a high expression level and average Cq scores of 17.66, 18.18, 19.35, and 19.68, respectively. Group-II included *RPS9*, *GAPDH,* and *ACTB* that displayed an intermediate expression level with average Cq scores of 20.33, 21.69, and 22.27, respectively. Group-III included *UXT*, *HPRT1,* and *HMBS* with the least expression and average Cq scores of 24.76, 26.18, and 27.86, respectively (Table 2). Considering the distribution of average raw Cq scores and interquartile range, *EEF1A1* exhibited the lowest coefficient of variations (lowest variability across samples). On the other hand, the *HMBS* gene showed the highest coefficient of variation. Based on this parameter, *EEF1A1*, *B2M*, *RPS15,* and *RPL4* RGs were most stable while *UXT*, *HPRT1,* and *HMBS* genes were the least stable across the combined dataset (Figure 2).

**TABLE 2:** The average raw Ct values of individual RGs in cattle populations adapted to cold arid high-altitude hypoxia (LAC, HFX, JYC) and hot arid normoxia environments (SAC, KFC, HFC).

	S.no	Animal	*GAPDH*	*RPL4*	*EEF1A1*	*RPS9*	*HPRT11*	*UXT*	*HMBS*	*B2M*	*RPS15*	*ACTB*
Cold Arid Hypoxia group	1	LAC1	22.17	21.56	18.81	22.40	30.18	26.07	29.44	19.36	19.51	24.35
	2	LAC2	21.87	20.06	17.32	20.15	28.38	23.86	27.99	18.20	19.79	22.88
	3	LAC3	22.08	19.67	16.90	19.98	27.95	24.18	27.63	18.72	19.55	23.52
	4	LAC4	22.30	20.15	17.26	19.87	28.14	24.55	27.64	18.57	19.57	24.66
	5	LAC5	22.53	19.61	17.09	20.04	28.87	24.68	27.59	18.75	19.21	22.64
	6	LAC6	22.07	19.75	17.23	20.41	29.69	24.51	27.79	17.74	19.45	24.90
	7	HFX1	21.52	19.35	16.90	19.73	27.46	24.30	27.80	17.61	18.80	21.33
	8	HFX2	21.16	19.40	17.21	20.13	27.31	24.83	27.94	17.18	19.09	20.65
	9	HFX3	21.64	19.71	17.12	20.07	27.71	24.41	27.77	17.82	19.12	21.16
	10	HFX4	21.66	19.76	17.00	20.13	28.55	23.46	27.81	17.66	18.93	21.71
	11	HFX5	21.90	19.78	17.48	20.08	27.83	26.76	27.92	17.81	19.38	22.36
	12	JYC1	21.71	19.39	17.25	20.13	28.40	25.27	27.77	17.64	19.16	21.82
	13	JYC2	21.41	20.10	17.79	20.59	27.80	26.42	27.87	17.72	19.74	22.03
	14	JYC3	21.44	19.34	17.21	20.53	27.43	26.31	26.89	17.82	19.43	27.44
	15	JYC4	22.24	20.58	18.34	21.14	28.26	29.62	28.56	18.85	20.24	22.93
Hot Arid Normoxia group	16	SAC1	21.60	20.38	18.34	20.40	24.43	27.86	28.21	18.09	19.83	21.60
	17	SAC2	21.87	20.18	18.36	20.63	24.95	23.99	27.97	18.04	19.39	21.09
	18	SAC3	22.96	20.04	18.70	20.73	24.09	24.36	28.57	18.91	19.72	23.71
	19	SAC4	22.49	20.65	18.78	20.84	24.64	24.63	28.68	18.29	20.11	22.49
	20	SAC5	22.23	20.80	19.10	21.00	24.91	24.90	28.90	18.71	20.49	22.04
	21	KFC1	22.07	19.84	18.18	20.73	24.58	24.25	27.81	18.10	19.88	21.79
	22	KFC2	21.63	19.73	19.32	20.77	24.44	24.17	27.51	17.75	19.63	21.18
	23	KFC3	21.45	19.24	17.66	19.89	23.60	24.23	27.31	17.73	18.97	21.20
	24	KFC4	21.39	18.73	17.51	19.92	23.71	23.81	26.99	17.57	18.39	21.41
	25	KFC5	21.74	19.65	17.94	20.65	24.40	24.30	28.04	17.95	19.54	21.51
	26	HFC1	19.47	17.07	15.49	18.01	21.51	21.83	26.62	15.69	17.02	18.73
	27	HFC2	22.44	20.56	19.27	21.35	25.41	25.16	28.58	20.29	20.32	22.72
	28	HFC3	19.08	17.03	15.36	17.81	21.59	21.53	24.87	17.95	16.73	18.12
	29	HFC4	20.76	18.38	16.84	21.11	24.78	24.53	29.61	20.04	20.43	24.27
	30	HFC5	21.95	19.90	18.26	20.73	24.61	24.25	27.72	18.96	19.21	21.78
Average			21.7	19.7	17.7	20.3	26.2	24.8	27.9	18.2	19.4	22.3

LAC, ladakhi cattle; HFX, holstein friesian cross; JYC, jersey cattle; SAC, sahiwal cattle; KFC, karan fries cattle; HFC, holstein friesian cattle.

Similar to the combined dataset, in cold arid as well as hot arid normoxia groups, *EEF1A1* showed the highest maximum and *HMBS* showed the least expression level. The average Cq values of individual RGs in cold arid hypoxia and hot arid normoxia groups are summarized in Supplementary Tables S1,S2 and Supplementary Figures S3,S4 respectively.

### Selection of Reference Genes by geNorm Analysis

**FIGURE 3:**
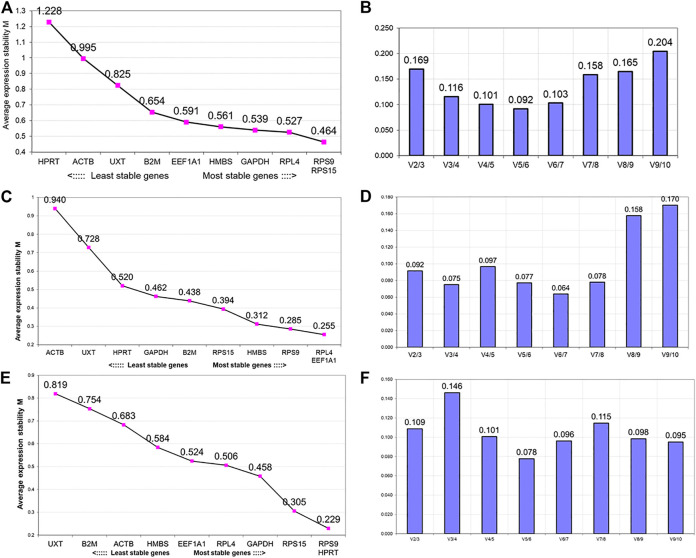
GeNorm analysis for ranking of genes based on average expression stability measure (M value) and pair-wise variation (Vn/Vn+ 1) between the normalisation factors NFn and NFn + 1 to determine the optimal number of reference genes. Analysis in the combined group (cold arid and hot arid) (**A** and **B**, respectively), cold arid hypoxia group (**C** and **D**, respectively), and hot arid normoxia group (**E** and **F**, respectively).

**FIGURE 4:**
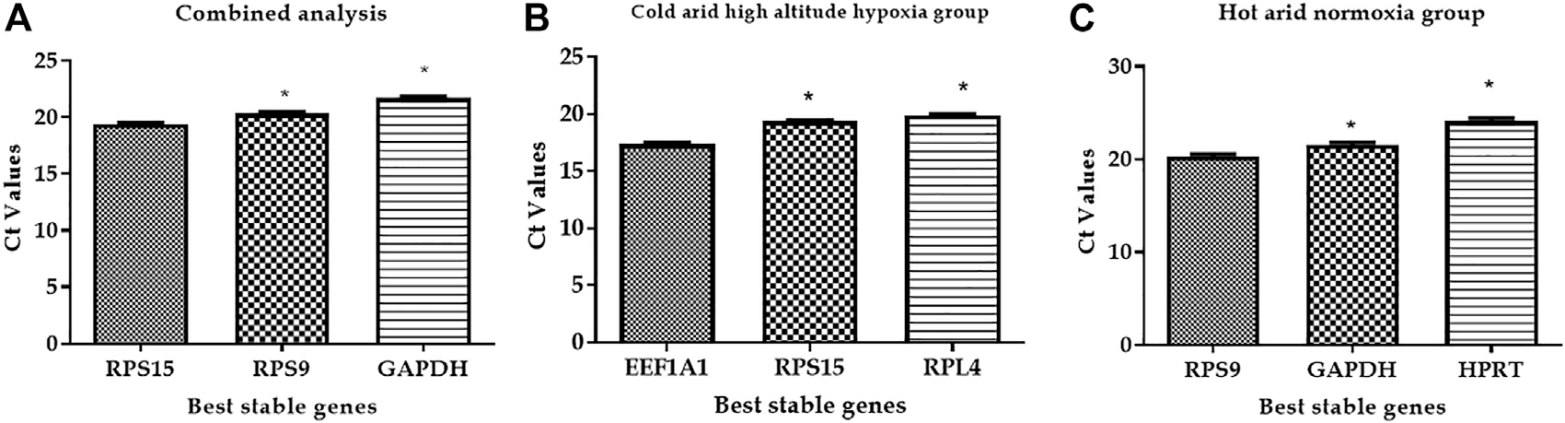
Expression stability of the three best stable RGs. **(A)** Combined group (cold arid and hot arid). **(B)** cold arid high-altitude hypoxia group, and **(C)** hot arid normoxia group.

The first analysis to determine the expression stability of 10 candidate RGs in PBMCs of heifer cows from the hypoxia cold arid group (LAC, JYC, and HFX) and normoxia hot arid group (SAC, KFC, and HFC) was based on the geNorm algorithm. The expression stability of individual RGs was evaluated first by combining RT-qPCR data for all 30 PBMCs samples (cold arid hypoxia and hot arid normoxia groups). All the RGs in combined group analysis showed stable expression with a stability index within the acceptable range (<M value < 1.5). In the combined analysis, *RPS9* and *RPS15* showed the highest expression stability (*M* = 0.464), followed by *RPL4* (*M* = 0.527) and *GAPDH* (*M* = 0.539), whereas *HPRT1* was the least stable gene (*M* = 1.228). On the basis of average expression stability measure, RGs were arranged from most stable (lowest M value) to the least stable (highest M value): *RPS9 = RPS15* > *RPL4*> *GAPDH* > HMBS > EEF1A1> *B2M* > *UXT* > *ACTB* > *HPRT1* (Figure 3A). Since the samples were from two distinct altitudes, we tried to determine the optimal number of RGs on the basis of pair-wise variation (Vn/n+1). The V values were calculated in different combinations: V2/V3, V3/V4, V4/V5, V5/V6 by adding the third, fourth, fifth, and sixth less stable genes, respectively. The V values for V2/V3 (0.169), V3/V4 (0.116), V4/V5 (0.101), V5/V6 (0.092), and V6/V7 (0.103) were either > or < the threshold of 0.15 (Figure 3B). It is generally considered that when Vn/n+1 is < 0.15 (threshold value), inclusion of an additional gene is not required for calculating the normalisation factor. In the combined dataset, combination V3/V4 resulted in a Vn/n+1 value < 0.116 which was less than the cut off value of 0.15 suggesting that the first three most stable RGs will be sufficient for accurate normalisation of RT-qPCR data. Therefore, based on M (stability) and V values (pair-wise variation) derived from geNorm analysis, the most stable RGs for the combined dataset were *RPS9, RPS15,* and *RPL4* (Figure 4 and Table 3).

**TABLE 3:** Overall ranking of the best suitable reference genes in combined (cold arid and hot arid groups), Cold arid hypoxia group, and hot arid normoxia group analysis.

Group	Ranking	geNorm	NormFinder	BestKeeper		ReFinder
		M value	Stability value	STDEV	coff. of corr (r)	
Combined analysis	1	RPS9/RPS15 (0.464)	RPL4 (0.282)	GAPDH (0.52)	RPS9 (0.901)	RPS9 (1.41)
	2	RPL4 (0.527)	RPS9 (0.289)	HMBS (0.55)	RPL4 (0.894)	RPS15 (1.86)
	3	GAPDH (0.539)	RPS15 (0.292)	RPS15 (0.55)	RPS15 (0.890)	GAPDH (2.83)
	4	HMBS (0.561)	GAPDH (0.302)	RPS9 (0.58)	GAPDH (0.849)	RPL4 (3.41)
	5	EEF1A1 (0.591)	HMBS (0.382)	RPL4 (0.62)	HMBS (0.795)	HMBS (3.98)
	6	B2M (0.654)	B2M (0.432)	B2M (0.63)	ACTB (0.748)	EEF1A1 (6.48)
	7	UXT (0.825)	EEF1A1 (0.532)	EEF1A1 (0.77)	UXT (0.721)	B2M (6.48)
	8	ACTB (0.995)	UXT (0.605)	UXT (1.03)	EEF1A1 (0.704)	UXT (8)
	9	HPRT1 (1.228)	ACTB (0.737)	ACTB (1.29)	B2M (0.649)	ACTB (9)
	10	—	HPRT1 (1.288)	HPRT1 (2.08)	HPRT1 (0.640)	HPRT1 (10)
Cold arid hypoxia group	1	RPL4/EEF1A1 (0.255)	RPS15 (0.140)	RPS15 (0.28)	EEF1A1 (0.865)	EEF1A1 (1.41)
	2	RPS9 (0.285)	EEF1A1 (0.140)	GAPDH (0.32)	RPS9 (0.858)	RPL4 (2.11)
	3	HMBS (0.312)	RPS9 (0.155)	HMBS (0.32)	RPL4 (0.846)	RPS15 (3.16)
	4	RPS15 (0.394)	RPL4 (0.200)	EEF1A1 (0.38)	B2M (0.781)	RPS9 (3.57)
	5	B2M (0.438)	B2M (0.233)	RPL4 (0.41)	RPS15 (0.717)	HMBS (3.87)
	6	GAPDH (0.462)	GAPDH (0.259)	RPS9 (0.44)	UXT (0.639)	GAPDH (5.45)
	7	HPRT1 (0.520)	HMBS (0.320)	B2M (0.52)	HMBS (0.619)	B2M (5.96)
	8	UXT (0.728)	HPRT1 (0.389)	HPRT1 (0.60)	HPRT1 (0.612)	HPRT1 (8)
	9	ACTB (0.940)	UXT (0.685)	UXT (1.17)	GAPDH (0.571)	UXT (9)
	10	—	ACTB (0.808)	ACTB (1.34)	ACTB (0.571)	ACTB (10)
Hot arid normoxia group	1	HPRT1/RPS9 (0.229)	RPS15 (0.107)	GAPDH (0.74)	RPS9 (0.978)	RPS9 (1.32)
	2	RPS15 (0.305)	RPS9 (0.134)	B2M (0.74)	RPS15 (0.971)	HPRT1 (2.71)
	3	RPL4 (0.458)	HPRT1 (0.136)	RPS9 (0.75)	HPRT1 (0.962)	GAPDH (2.83)
	4	GAPDH (0.506)	HMBS (0.200)	HMBS (0.79)	RPL4 (0.905)	RPS15 (3.03)
	5	EEF1A1 (0.524)	GAPDH (0.225)	UXT (0.80)	GAPDH (0.904)	HMBS (5.38)
	6	HMBS (0.584)	RPL4 (0.225)	HPRT1 (0.81)	EEF1A1 (0.889)	RPL4 (6.06)
	7	ACTB (0.683)	EEF1A1 (0.238)	RPS15 (0.83)	HMBS (0.879)	B2M (6.18)
	8	B2M (0.754)	UXT (0.292)	EEF1A1 (0.91)	ACTB (0.879)	EEF1A1 (6.96)
	9	UXT (0.819)	ACTB (0.307)	RPL4 (0.93)	UXT (0.790)	UXT (8.41)
	10	—	B2M (0.363)	ACTB (1.04)	B2M (0.715)	ACTB (8.46)

In addition to combined analysis, the geNorm tool was used to evaluate the expression stability of individual RGs separately in the cold arid hypoxia group (LAC, JYC, and HFX) and hot arid normoxia group (SAC, KFC, and HFC). The M values for all the 10 RGs in the hypoxia cold arid group were <1.5. The ranking of RGs in order of expression stability within this group were: *RPL4* = *EEF1A1* >*RPS9*> *HMBS > RPS15* > *B2M* > *GAPDH* > *HPRT11* > *UXT* > *ACTB* (Figure 3C; Table 3). *RPL4* and *EEF1A1* showed the highest expression stability (*M* = 0.255) followed by *RPS9* (*M* = 0.285) and *HMBS* (*M* = 0.312), whereas *ACTB* was least stable (*M* = 0.940). The pair-wise variation analysis for different combinations of RGs was well below the threshold value of 0.15. The results obtained for V2/3 (0.092), V3/4 (0.075), V4/5 (0.097), and V5/6 (0.077) combinations indicated that *RPL4* and *EEF1A1* would provide the most accurate normalisation (Figure 3D). The expression stability of the three best RGs in combined analysis is shown in Figure 4B.

In the hot arid normoxia group (SAC, KFC, and HFC), the ranking of RGs in order of expression stability were: *HPRT1* = *RPS9*> *RPS15* > *GAPDH* > *RPL4*> *EEF1A1*> *HMBS* > *ACTB* > *B2M* > *UXT* (Figure 3E; Table 3). The M values for all the 10 RGs were <1.5. *HPRT1* (*M* = 0.229) and *RPS9* (*M* = 0.229) showed the highest stability followed by *RSP15* (*M* = 0.305) whereas *UXT* was least stable (*M* = 0.819). The results for V2/3 (0.109), indicated that *RPS9* and *HPRT1* would provide reliable normalisation in PBMCs of the hot arid normoxia group (Figure 3F). The results of geNorm analysis for the three groups are summarized in Table 3. Overall, combined and group-wise analysis implied that *RPL4, RPS9*, *RPS15,* and *EEF1A1* were the most stably expressed RGs (Figure 4C).

### Selection of Reference Genes by NormFinder Analysis

**FIGURE 5:**
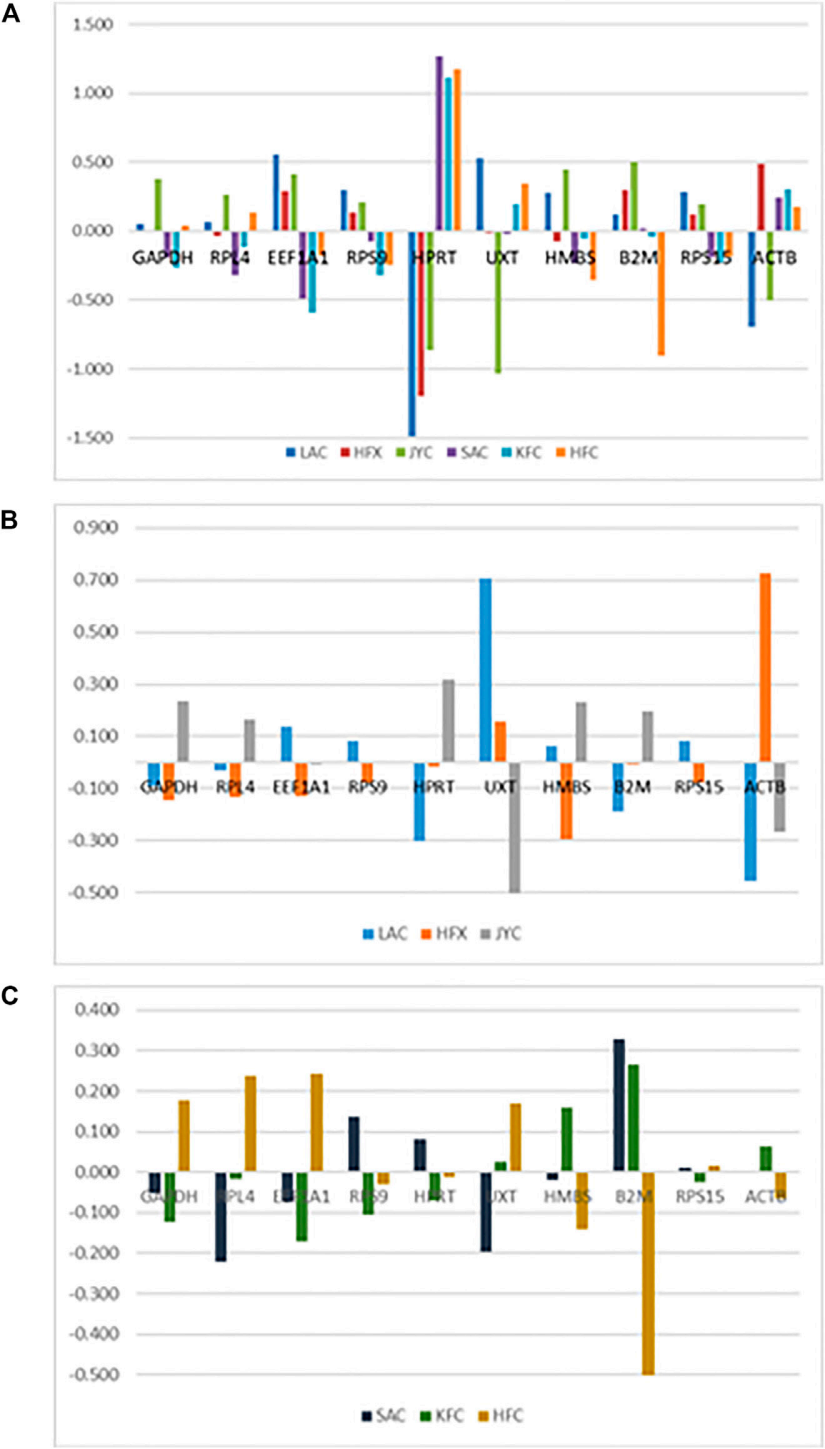
Inter-group variation analysis of RGs **(A)** Combined group (cold arid and hot arid). **(B)** cold arid hypoxia group, and **(C)** hot arid normoxia group.

In the NormFinder-based intergroup (combined) analysis covering both the conditions and all samples (cold arid hypoxia and hot arid normoxia), *RPL4, RPS9,* and *RPS15* were found to be most stable with stability values of 0.282, 0.289, and 0.292 respectively. On the other hand, *UXT*, *ACTB,* and *HPRT1* were least stable with stability values of 0.605, 0.737, and 1.288, respectively (Table 3). The graph showing intragroup variation of RGs in the combined dataset is shown in Figure 5A. Based on stability values, the RGs were ranked as *RPL4*> *RPS9*> *RPS15* > *GAPDH* > *HMBS* > *B2M* > *EEF1A1*> *UXT* > *ACTB* > *HPRT1*. Within the cold arid hypoxia group, *RPS15*, *EEF1A1,* and *RPS9* were the most stable RGs with stability values of 0.140, 0.140, and 0.155, respectively, whereas, *ACTB* was the least stable gene with the highest variability value of 0.808 (Table 3). The graph showing intragroup variation analysis of RGs is shown in Figure 5B. The RGs were ranked as *RPS15> EEF1A1> RPS9> RPL4> B2M > GAPDH > HMBS > HPRT1>UXT > ACTB.*

Within the hot arid normoxia group, NormFinder analysis identified *RPS15*, *RPS9*, and *HPRT1* as the three most stable RGs with stability values of 0.107, 0.134, and 0.136, respectively (Table 3). The graph showing intragroup variation analysis of RGs in the combined dataset is shown in Figure 5C. Based on stability values, the RGs were ranked as *RPS15* > *RPS9*> *HPRT1*> *HMBS* > *GAPDH* > *RPL4*> *EEF1A1*> *UXT* > *ACTB* > *B2M*. Overall, there was a good agreement in geNorm and NormFinder outcomes for all the datasets (Table 3).

### Selection of Reference Genes by BestKeeper Analysis

The descriptive statistics for BestKeeper analysis of the combined dataset are shown in Table 4. The analysis suggested stable expression (SD < 1) for the majority of genes: *GAPDH* (CV ± SD = 2.40 ± 0.52), *HMBS* (CV ± SD = 1.97 ± 0.55), *RPS15* (CV ± SD = 2.85 ± 0.55)*, RPS9* (CV ± SD = 2.85 ± 0.58), *RPL4* (CV ± SD = 3.15 ± 0.62), *B2M* (CV ± SD = 3.46 ± 0.63), and *EEF1A1* (CV ± SD = 4.35 ± 0.77). Only three genes, *UXT* (CV ± SD = 4.18 ± 1.03), *ACTB* (CV ± SD = 5.81 ± 1.29), and *HPRT1* (CV ± SD = 7.93 ± 2.07) showed SD > 1 and hence were considered unacceptable as RGs. Additionally, the BestKeeper software was used to measure the inter-gene relation amongst the 10 RGs in the combined dataset by calculating Pearson correlation coefficient (*r*) values. Several RG pairs *GAPDH/RPL4* (*r* = 0.861) *RPS9/RPS15* (*r* = 0.849), *RPS9/HMBS* (*r* = 0.847), *RPL4/RPS9* (*r* = 0.834), and *HMBS/RPS5* (*r* = 0.832) showed high correlation coefficients (Table 5). The RG pairs with high *r* values suggested that their expression pattern in PBMCs of cattle populations from two distinct climates and altitudes are more or less similar to each other. Further, the BestKeeper index (BI) calculated for each gene was used to estimate the correlation value between BI and each RG. As shown in Table 5, the best correlation values were obtained for *RPS9* (*r* = 0.901), *RPL4* (*r* = 0.894), *RPS15* (*r* = 0.890), and *GAPDH* (*r* = 0.849). The high correlation values strongly suggested their suitability as reliable RGs in the experimental set up of the study.

**TABLE 4:** Combined (cold arid and hot arid groups) analysis of parameters-based quantitative cycling points (CP) for 10 candidate RGs

	*GAPDH*	*RPL4*	*EEF1A1*	*RPS9*	*HPRT1*	*UXT*	*HMBS*	*B2M*	*RPS15*	*ACTB*
N	30	30	30	30	30	30	30	30	30	30
geo Mean [CP]	21.68	19.66	17.64	20.31	26.09	24.72	27.85	18.16	19.34	22.2
ar Mean [CP]	21.69	19.68	17.67	20.33	26.19	24.77	27.86	18.18	19.35	22.27
min [CP]	19.08	17.03	15.36	17.81	21.51	21.53	24.87	15.69	16.73	18.12
max [CP]	22.96	21.56	19.32	22.4	30.18	29.62	29.61	20.29	20.49	27.44
std dev [± CP]	0.52	0.62	0.77	0.58	2.08	1.03	0.55	0.63	0.55	1.29
CV [% CP]	2.4	3.15	4.34	2.85	7.93	4.18	1.97	3.46	2.85	5.81
min [x-fold]	−6.06	−6.18	−4.86	−5.67	−23.84	−9.14	−7.87	−5.56	−6.09	−16.89
max [x-fold]	2.43	3.74	3.2	4.25	17.08	29.82	3.39	4.37	2.23	37.85
std dev [± x-fold]	1.44	1.54	1.7	1.5	4.22	2.05	1.46	1.55	1.47	2.45

N, number of samples; geo Mean [CP], geometric mean of CP; ar Mean [CP], arithmetic mean of CP; min [CP] and max [CP], extreme values of CP; Std dev [±CP], standard deviation of the CP; CV [%CP], coefficient of variation expressed as a percentage on the CP, values; min [x-fold] and max [x-fold], extreme values of expression levels expressed as absolute x-fold over or under the coefficient; std dev[±x-fold], standard deviation of the absolute regulation coefficients.

**TABLE 5:** Combined (cold arid and hot arid groups) analysis of repeated pair-wise correlation amongst genes with the BestKeeper index.

	GAPDH	RPL4	EEF1A1	RPS9	HPRT1	UXT	HMBS	B2M	RPS15	ACTB
RPL4	0.861	—	—	—	—	—	—	—	—	—
*p*-value	0.001	—	—	—	—	—	—	—	—	—
*EEF1A1*	0.725	0.817	—	—	—	—	—	—	—	—
*p*-value	0.001	0.001	—	—	—	—	—	—	—	—
*RPS9*	0.722	0.834	0.822	—	—	—	—	—	—	—
*p*-value	0.001	0.001	0.001	—	—	—	—	—	—	—
*HPRT1*	0.489	0.523	0.041	0.39	—	—	—	—	—	—
*p*-value	0.006	0.003	0.829	0.033	—	—	—	—	—	—
*UXT*	0.469	0.614	0.468	0.586	0.457	—	—	—	—	—
*p*-value	0.009	0.001	0.009	0.001	0.011	—	—	—	—	—
*HMBS*	0.639	0.714	0.636	0.847	0.351	0.5	—	—	—	—
*p*-value	0.001	0.001	0.001	0.001	0.057	0.005	—	—	—	—
B2M	0.509	0.494	0.491	0.66	0.178	0.307	0.589	—	—	—
*p*-value	0.004	0.006	0.006	0.001	0.348	0.099	0.001	—	—	—
*RPS15*	0.769	0.787	0.741	0.849	0.366	0.615	0.831	0.643	—	—
*p*-value	0.001	0.001	0.001	0.001	0.047	0.001	0.001	0.001	—	—
*ACTB*	0.569	0.478	0.270	0.584	0.57	0.458	0.458	0.483	0.609	—
*p*-value	0.001	0.008	0.149	0.001	0.001	0.011	0.011	—	—	—
BestKeeper vs.	GAPDH	RPL4	EEF1A1	RPS9	HPRT1	UXT	HMBS	B2M	RPS15	ACTB
coeff. of corr. [r]	0.849	0.894	0.704	0.901	0.640	0.721	0.795	0.649	0.890	0.748
*p*-value	0.001	0.001	0.001	0.001	0.001	0.001	0.001	0.001	0.001	0.001

The BestKeeper analysis for the cold arid hypoxia group and hot arid normoxia group was also conducted separately. In the cold arid hypoxia group, the following 8 RGs showed lower a coefficient of variation (CV) and standard deviation (SD): *RPS15* (CV ± SD = 1.44 ± 0.28), *HMBS* (CV ± SD = 1.14 ± 0.32), *GAPDH* (CV ± SD = 1.46 ± 0.32), *EEF1A1* (CV±SD = 2.18 ± 0.38), *RPL4* (CV ± SD = 2.04 ± 0.41), *RPS9* (CV ± SD = 2.15 ± 0.44), *B2M* (CV ± SD = 2.85 ± 0.52), and *HPRT1* (CV ± SD = 2.12 ± 0.60) (Table 6). The remaining two RGs showed high SD and CV values: *ACTB* (CV ± SD = 5.85 ± 1.34) and *UXT* (CV ± SD = 4.62 ± 1.17) and were unstable. The inter-gene relation showed very high correlation coefficient values (*r*) for *EEF1A1*/*RPS9* (*r* = 0.93), *EEF1A1*/*RPL4* (*r* = 0.90), and *RPL4*/*RPS9* (*r* = 0.86) (Table 7). Further, the coefficient of correlation analysis (*r*) of individual RGs with the BestKeeper index resulted in high values for *EEF1A1* (*r* = 0.865), *RPS9* (*r* = 0.858), and *RPL4* (*r* = 0.846). These results strongly suggest that *EEF1A1*, *RPS9,* and *RPL4* with low CV, low SD, and high coefficient of correlation to the BestKeeper index should be the ideal panel for the cold arid high-altitude hypoxia group.

**TABLE 6:** Parameters-based quantitative cycling points (CP) for 10 RGs in PBMCs of cattle populations adapted to cold arid high-altitude hypoxia conditions.

	GAPDH	RPL4	EEF1A1	RPS9	HPRT1	UXT	HMBS	B2M	RPS15	ACTB
N	15	15	15	15	15	15	15	15	15	15
geo Mean [CP]	21.84	19.87	17.39	20.35	28.25	25.24	27.89	18.09	19.39	22.9
ar Mean [CP]	21.85	19.88	17.39	20.36	28.26	25.28	27.89	18.1	19.4	22.96
min [CP]	21.16	19.34	16.9	19.73	27.31	23.46	26.89	17.18	18.8	20.65
max [CP]	22.53	21.56	18.81	22.4	30.18	29.62	29.44	19.36	20.24	27.44
std dev [+/- CP]	0.32	0.41	0.38	0.44	0.6	1.17	0.32	0.52	0.28	1.34
CV [% CP]	1.46	2.04	2.18	2.15	2.12	4.63	1.14	2.85	1.44	5.85
min [x-fold]	−1.61	−1.45	−1.4	−1.54	−1.92	−3.43	−2	−1.88	−1.51	−4.75
max [x-fold]	1.61	3.22	2.68	4.14	3.8	20.83	2.93	2.42	1.8	23.31

N, number of samples, geo Mean [CP], geometric mean of CP; ar Mean [CP], arithmetic mean of CP; min [CP] and max [CP], extreme values of CP; Std dev [±CP], standard deviation of the CP; CV [%CP], coefficient of variation expressed as a percentage on the CP, values; min [x-fold] and max [x-fold], extreme values of expression levels expressed as absolute x-fold over or under the coefficient; std dev[±x-fold], standard deviation of the absolute regulation coefficients.

**TABLE 7:** Repeated pair-wise correlation amongst genes with the BestKeeper index in cattle populations adapted to cold arid high-altitude hypoxia conditions.

	GAPDH	RPL4	EEF1A1	RPS9	HPRT1	UXT	HMBS	B2M	RPS15	ACTB
RPL4	0.458	—	—	—	—	—	—	—	—	—
*p*-value	0.086	—	—	—	—	—	—	—	—	—
EEF1A1	0.261	0.896	—	—	—	—	—	—	—	—
*p*-value	0.348	0.001	—	—	—	—	—	—	—	—
RPS9	0.213	0.856	0.925	—	—	—	—	—	—	—
*p*-value	0.446	0.001	0.001	—	—	—	—	—	—	—
HPRT1	0.617	0.635	0.489	0.612	—	—	—	—	—	—
*p*-value	0.014	0.011	0.065	0.015	—	—	—	—	—	—
UXT	0.128	0.401	0.702	0.531	−0.031	—	—	—	—	—
*p*-value	0.65	0.139	0.004	0.042	0.913	—	—	—	—	—
HMBS	0.285	0.866	0.831	0.78	0.589	0.339	—	—	—	—
*p*-value	0.303	0.001	0.001	0.001	0.021	0.216	—	—	—	—
B2M	0.819	0.739	0.566	0.568	0.551	0.283	0.524	—	—	—
*p*-value	0.001	0.002	0.028	0.027	0.033	0.306	0.045	—	—	—
RPS15	0.401	0.554	0.6	0.443	0.175	0.647	0.302	0.537	—	—
*p*-value	0.138	0.032	0.018	0.098	0.532	0.009	0.274	0.039	—	—
ACTB	0.311	0.201	0.177	0.324	0.286	0.182	−0.187	0.378	0.384	—
*p*-value	0.259	0.473	0.529	0.239	0.302	0.516	0.504	0.165	0.158	—
BestKeeper vs.	GAPDH	RPL4	EEF1A1	RPS9	HPRT1	UXT	HMBS	B2M	RPS15	ACTB
coeff. of corr. [r]	0.569	0.846	0.866	0.858	0.613	0.639	0.618	0.782	0.715	0.571
*p*-value	0.027	0.001	0.001	0.001	0.015	0.01	0.014	0.001	0.003	0.026

The BestKeeper analysis for the hot arid normoxia group also resulted in several RGs with SD < 1 (Table 8). The following nine RGs: *GAPDH* (CV ± SD = 3.44 ± 0.74), *B2M* (CV ± SD = 4.07 ± 0.74), *HMBS* (CV ± SD = 3.47 ± 0.96), *RPS 9* (CV ± SD = 2.85 ± 0.79), *UXT* (CV ± SD = 3.28 ± 0.80), *HPRT1* (CV ± SD = 3.35 ± 0.81), *RPS15* (4.30 ± 0.83), *EEF1A1* (CV ± SD = 5.09 ± 0.91), and *RPL4* (CV ± SD = 4.75 ± 0.93) showed low SD and CV values. Whereas *ACTB* (CV ± SD = 54.82 ± 1.04) showed SD > 1 and hence was considered unstable.

**TABLE 8:** Parameters-based quantitative cycling points (CP) for 10 RGs in cattle populations adapted to hot arid normoxia conditions.

	GAPDH	RPL4	EEF1A1	RPS9	HPRT1	UXT	HMBS	B2M	RPS15	ACTB
N	15	15	15	15	15	15	15	15	15	15
geo Mean [CP]	21.52	19.44	17.9	20.28	24.08	24.22	27.8	18.24	19.28	21.52
ar Mean [CP]	21.54	19.48	17.94	20.3	24.11	24.25	27.83	18.27	19.31	21.58
min [CP]	19.08	17.03	15.36	17.81	21.51	21.53	24.87	15.69	16.73	18.12
max [CP]	22.96	20.8	19.32	21.35	25.41	27.86	29.61	20.29	20.49	24.27
std dev [+/- CP]	0.74	0.93	0.91	0.75	0.81	0.8	0.79	0.74	0.83	1.04
CV [% CP]	3.44	4.75	5.09	3.67	3.35	3.28	2.85	4.07	4.3	4.82
min [x-fold]	−5.41	−5.33	−5.81	−5.53	−5.95	−6.43	−7.64	−5.86	−5.85	−10.56
max [x-fold]	2.72	2.56	2.68	2.1	2.51	12.51	3.5	4.14	2.32	6.73
std dev [+/- x-fold]	1.67	1.9	1.88	1.68	1.75	1.74	1.73	1.67	1.78	2.05

N, number of samples; geo Mean [CP], geometric mean of CP; ar Mean [CP], arithmetic mean of CP; min [CP] and max [CP], extreme values of CP; Std dev [±CP], standard deviation of the CP; CV [%CP], coefficient of variation expressed as a percentage on the CP, values; min [x-fold] and max [x-fold], extreme values of expression levels expressed as absolute x-fold over or under the coefficient; std dev[±x-fold], standard deviation of the absolute regulation coefficients.

The inter-gene relation showed a strong correlation coefficient (*r*) for *GAPDH*/*RPL4* (*r* = 0.930), *EEF1A1*/RPL4 (*r* = 0.951), *RPS9/HPRT11* (*r* = 0.981), *RPS15/HMBS* (*r* = 0.923), *HMBS/ACTB* (*r* = 0.908), *HPRT11/RPL4* (*r* = 874), *HPRT11/EEF1A1* (*r* = 861), *HMBS*/*RPS9* (*r* = 0.874), and *RPS9*/*RPS15* (*r* = 0.965) (Table 9). The highest correlation coefficient of individual RGs with the BestKeeper index was observed for *RPS9* (*r* = 0.978), *RPS15* (0.971), *HPRT1* (*r* = 0.962), *RPL4* (0.905), and *GAPDH* (*r* = 0.904) (Table 9). The high correlation coefficient of these genes suggested their suitability as RGs in the hot arid normoxia group.

**TABLE 9:** Repeated pair-wise correlation amongst genes with the BestKeeper index of cattle populations adapted to hot arid normoxia conditions.

	GAPDH	RPL4	EEF1A1	RPS9	HPRT1	UXT	HMBS	B2M	RPS15	ACTB
RPL4	0.93	—	—	—	—	—	—	—	—	—
*p*-value	0.001	—	—	—	—	—	—	—	—	—
EEF1A1	0.923	0.951	—	—	—	—	—	—	—	—
*p*-value	0.001	0.001	—	—	—	—	—	—	—	—
RPS9	0.869	0.852	0.865	—	—	—	—	—	—	—
*p*-value	0.001	0.001	0.001	—	—	—	—	—	—	—
HPRT1	0.841	0.874	0.861	0.981	—	—	—	—	—	—
*p*-value	0.001	0.001	0.001	0.001	—	—	—	—	—	—
UXT	0.64	0.758	0.678	0.706	0.726	—	—	—	—	—
*p*-value	0.01	0.001	0.005	0.003	0.002	—	—	—	—	—
HMBS	0.711	0.689	0.644	0.874	0.828	0.674	—	—	—	—
*p*-value	0.003	0.004	0.009	0.001	0.001	0.006	—	—	—	—
B2M	0.495	0.475	0.468	0.703	0.698	0.447	0.618	—	—	—
*p*-value	0.061	0.073	0.079	0.003	0.004	0.095	0.014	—	—	—
RPS15	0.819	0.842	0.822	0.965	0.943	0.762	0.923	0.69	—	—
*p*-value	0.001	0.001	0.001	0.001	0.001	0.001	0.001	0.004	—	—
ACTB	0.75	0.623	0.627	0.877	0.802	0.617	0.908	0.749	0.875	—
*p*-value	0.001	0.013	0.012	0.001	0.001	0.014	0.001	0.001	0.001	—
BestKeeper vs.	GAPDH	RPL4	EEF1A1	RPS9	HPRT1	UXT	HMBS	B2M	RPS15	ACTB
coeff. of corr. [r]	0.904	0.905	0.889	0.978	0.962	0.79	0.879	0.715	0.971	0.879
*p*-value	0.001	0.001	0.001	0.001	0.001	0.001	0.001	0.003	0.001	0.001

### Selection of Reference Genes by RefFinder Analysis

Additionally, the RefFinder algorithm was used to evaluate the comprehensive ranking of individual RGs in combined, cold arid hypoxia, and hot arid normoxia group datasets (Xie et al., 2012). In the combined dataset, RefFinder ranked *RPS9* (1.41), *RPS15* (1.86), and *GAPDH* (2.83) as the three most stable RGs, followed by *RPL4* (3.41), *HMBS* (3.98), *EEF1A1* (6.48), and *B2M* (6.48), whereas *UXT* (8.00), *ACTB* (9.00), and *HPRT1* (10.00) ranked as the three least stable RGs (Table 3). In the cold arid hypoxia group, RefFinder analysis ranked *EEF1A1* (1.41), *RPL4* (2.11), and *RPS15* (3.16) as the most stable RGs; while *HPRT11* (8.00), *UXT* (9.00), and *ACTB* (10.00) as the most unstable RGs. In the hot arid normoxia group, the analysis displayed *RPS9* (1.32), *HPRT1* (2.71), and *GAPDH* (2.83) as most stable while *EEF1A1* (6.96), *UXT* (8.41), and *ACTB* (8.46) as unstable RGs.

### Validation of Selected Reference Genes

**FIGURE 6:**
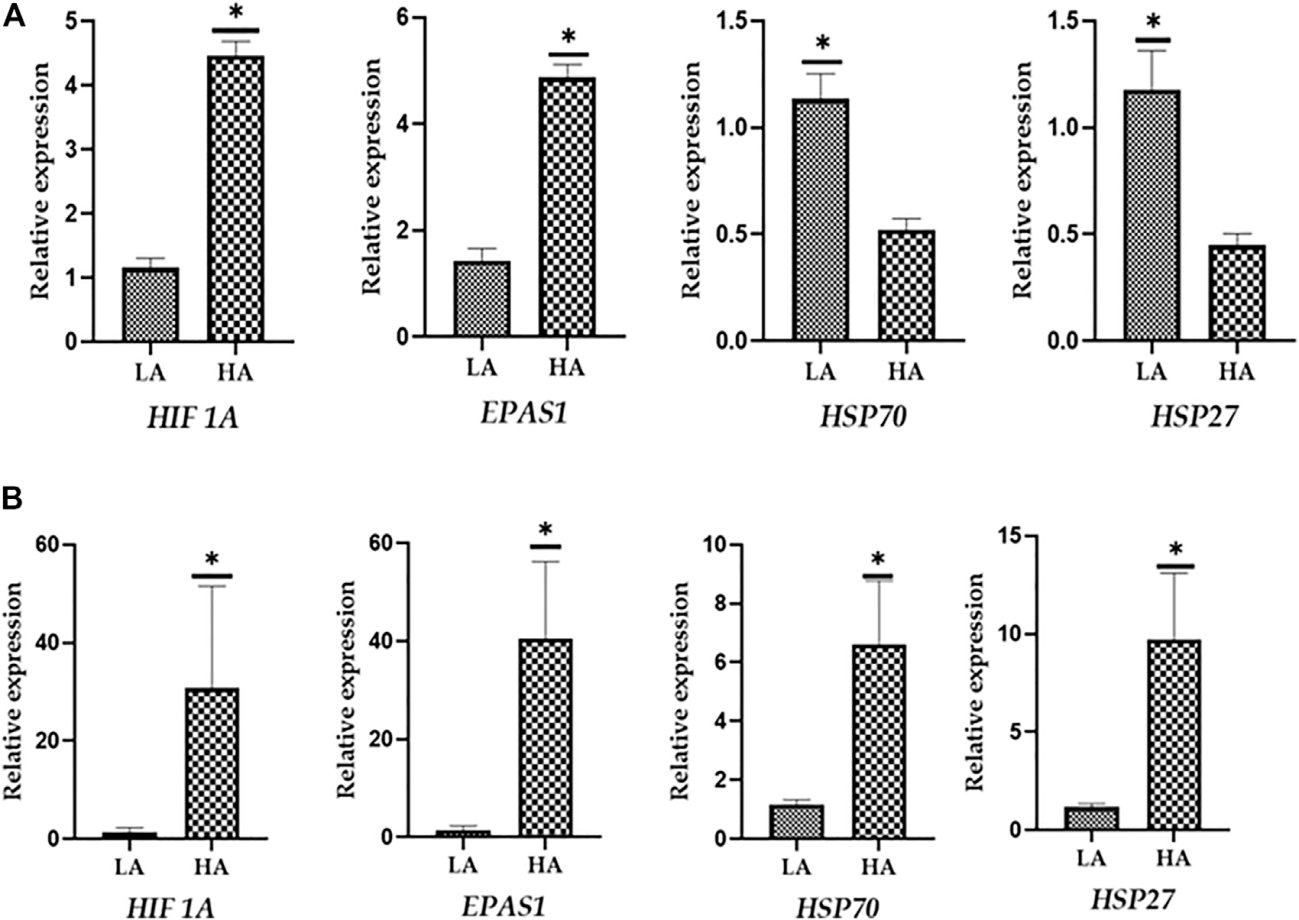
**(A) **Evaluation of best reference genes (*RPS19, RPS15,* and *GAPDH*) in normalising the target genes. **(B)** Evaluation of least stable reference genes (*HPRT* and *ACTB*) in normalising the target genes.

To evaluate the reliability of the best suitable and worst panel of RGs, a validation qPCR experiment was performed using some of the known candidate target genes associated with high-altitude hypoxia and heat stress response. The qPCR data for two target genes associated with hypoxia and high altitude such as HIF-alpha and *EPAS1* and two target genes associated with heat stress response such as *HSP70* and *HSP27* were generated in PBMC samples of high-altitude-adapted and tropically adapted cattle populations. As shown in Figure 6A, it is quite evident that the panel of best reference genes (*RPS19*, *RPS15,* and *GAPDH*) normalised the target gene data more accurately. We expected higher expression of high-altitude-associated genes in PBMCs of high-altitude cattle populations. On the other hand, genes related to heat stress response such as HSPs should have higher expression in PBMCs of cattle populations from the tropical region. As shown in Figure 6A, the expression of target genes such as HIF-*alpha* and *EPAS1* was higher in high-altitude (HA)-adapted cattle while genes like *HSP70* and *HSP27* were more expressed in low-altitude (LA) cattle breeds. Further, the relative expression, standard deviation (SD), and standard error (SE) of the four target genes also supported the high quality of the panel of reference genes used (Supplementary Table S3).

On the other hand, normalisation of same target genes with least stable genes (*HPRT* and *ACTB*) resulted in an unexpected pattern of expression. As shown in Figure 6B, HSPs showed higher expression in PBMCs of high-altitude cattle populations. Further, the use of least stable reference genes resulted in higher SD and SE values (Supplementary Table S4). Considering the above facts, it could be stated that the panel of RGs identified in the present study will be applicable for accurate normalisation of target genes in studies involving high and low-altitude cattle populations.

## Discussion

In an era of high-throughput platforms, quantitative PCR (qRT-PCR) is being employed as a most preferred tool to validate gene expression data. Even though qRT-PCR is the most sensitive technique, it suffers from several analytical variations like differences in the amount of starting material, RNA extraction, and efficiency of the reverse transcription process. To a great extent the effect of these non-biological variables can be nullified by normalising gene expression data by a panel of stable reference genes (RGs). As suggested in the “Minimum information for publication of Quantitative Real-time PCR Experiments” (MIQE) guideline (Bustin et al., 2009), accuracy in gene expression is largely governed by the availability of reliable RGs. At present there is no consensus for the set of reference gene(s) that can be used universally for the normalisation purposes. In the past, several studies have utilized RGs without proper validation or arbitrarily selected commonly used reference genes such as *ACTB* and *GAPDH*. Unfortunately, the use of such empirical RGs might not provide accurate normalisation and create doubt on the reliable estimation of gene expression. Moreover, many reports showed the variable expression of commonly used reference genes in different cells, tissue, and conditions (Kim and Yun 2011; Zhao et al., 2012; Thomas et al., 2014).

The major challenge in any biological experiment involving different tissues, cell types, disease state, and physiological and or developmental stages is the knowledge about appropriate RGs whose expression remains constant without any observable variations across samples (Bustin 2002; Radonić et al., 2004). It has been seen that a particular RG appropriate in one condition might have variable expression in another set of conditions. Thus, identification and validation of proper RGs is the prerequisite for any specific experimental condition. Our group has successfully identified a panel of appropriate RGs for various cellular types and experimental conditions involving zebu cattle and riverine buffaloes (Kishore et al., 2013; Sood et al., 2017; Lagah et al., 2019). The present study was also conducted on similar lines to identify suitable reference genes for cattle populations from diverse environmental conditions. The 10 RGs (RGs selected for the present work were part of our earlier studies, wherein these genes were evaluated for their suitability as normalisers in different cell types, cattle breeds, and experimental conditions (Kishore et al., 2013; Jatav et al., 2016; Kaur et al., 2018)). Multiple RGs are generally preferred over a single RG to reduce the experimental variation for more effective normalisation (Vandesompele et al., 2002; Huang et al., 2014; Engdahl et al., 2016; Xu et al., 2016). Hence, in the present study, an optimal number of genes to be used as normalisers was identified. The data were analysed to identify the most appropriate panel of RGs for the combined dataset (cold arid and hot arid) and separately for the cold arid high-altitude hypoxia group and the hot arid normoxia group. Based on overall analysis, *RPS9*, *RPS15*, and *GAPDH* were marked as the most stable RGs for combined data. In the cold arid hypoxia group; *RPL4*, *EEF1A1,* and *RPS15* were the most stable RGs, while in the hot arid normoxia cattle group, *RPS9*, *HPRT1*, and *GAPDH* were identified as the most stable RGs.

In the present analysis, *RPS9, RPS15,* and, to a certain extent, *RPL4* genes were identified as stable RGs in the three groups (combined dataset, cold arid hypoxia group, and hot arid normoxia group). Both *RPS9* and *RPS15* genes are part of the ribosomal component of the small 40S subunit while *RPL4* is a 60S ribosomal protein L4. In the past, these ribosomal genes were also identified as ideal reference genes in many studies involving the mammary gland of dairy cows (Bionaz and Loor 2007), mammary epithelial cells of native cows (Pradeep et al., 2014), different tissues of riverine buffaloes (Kaur et al., 2018), heat-stressed MECs of riverine buffaloes (Kapila et al., 2013), and PBMCs of native cows and riverine buffaloes (Kishore et al., 2013). It is known that these ribosomal proteins are highly conserved (Hsiao et al., 2001) and required by all life forms to synthesise new ribosomes. The unchanged expression of ribosomal proteins has also been reported in the red flour beetle (*T. castaneum*) during a fungal infection (Lu et al., 2018b). Similarly, stability of different ribosomal gene expressions has also been validated in several studies involving abiotic and biotic challenges (Ma et al., 2016; Yan et al., 2016; Lu et al., 2018a; Luo et al., 2018). In our study, the genes encoding ribosomal proteins also showed stabilised expression in PBMCs derived from different cattle types maintained at different altitudes. Based on current findings as well as previous studies, it could be concluded that ribosomal proteins exhibit higher expression stability and are good candidates as reference genes. *GAPDH*, the other stable RG in our study, has also been identified as an appropriate reference gene in several other studies (Lisowski et al., 2008; Yang et al., 2014; Engdahl et al., 2016; Jatav et al., 2016). Along with *GAPDH*, *EEF1A1* and *HPRT1* were the other genes that ranked amongst the first three most stable RGs in cold arid hypoxia and hot arid normoxia groups, respectively. *EEF1A1* regulates the enzymatic delivery of aminoacyl tRNAs to the ribosome while *HPRT11* catalyses the conversion of hypoxanthine to inosine monophosphate and guanine to guanosine monophosphate, and the regulates generation of purine nucleotides through the purine salvage pathway. To the best of our knowledge, this is the first study to assess the expression stability of 10 candidate RGs in cattle populations adapted to distinct altitudes.

## Conclusion

This study has identified an altitude-specific panel of RGs in PBMCs of cattle populations from hot arid normoxia and cold arid high-altitude hypoxic environments. In the cold arid hypoxia group, *RPL4*, *EEF1A1,* and *RPS15* RGs were the most stable, while in the hot arid normoxia group, *RPS9*, *HPRT1*, and *GAPDH* were identified as the most stable RGs. Further, the combined analysis resulted in identification of a panel of *RPS9, RPS15, and GAPDH* RGs that could act as a useful resource to unravel the accurate transcriptional profile of peripheral blood mononuclear cells of cattle populations adapted to tropical and high-altitude conditions.

## Data Availability

The original contributions presented in the study are included in the article/Supplementary Material, further inquiries can be directed to the corresponding author.
