# Supplementary material for: Identification of Internal Reference Genes in Peripheral Blood Mononuclear Cells of Cattle Populations Adapted to Hot Arid Normoxia and Cold Arid Hypoxia Environments
Source: Front Genet. 2022 Feb 1;12:730599. doi: 10.3389/fgene.2021.730599 (PMC8846307; doi:10.3389/fgene.2021.730599)
Supplement: Supplementary file 1 [file DataSheet1.docx]

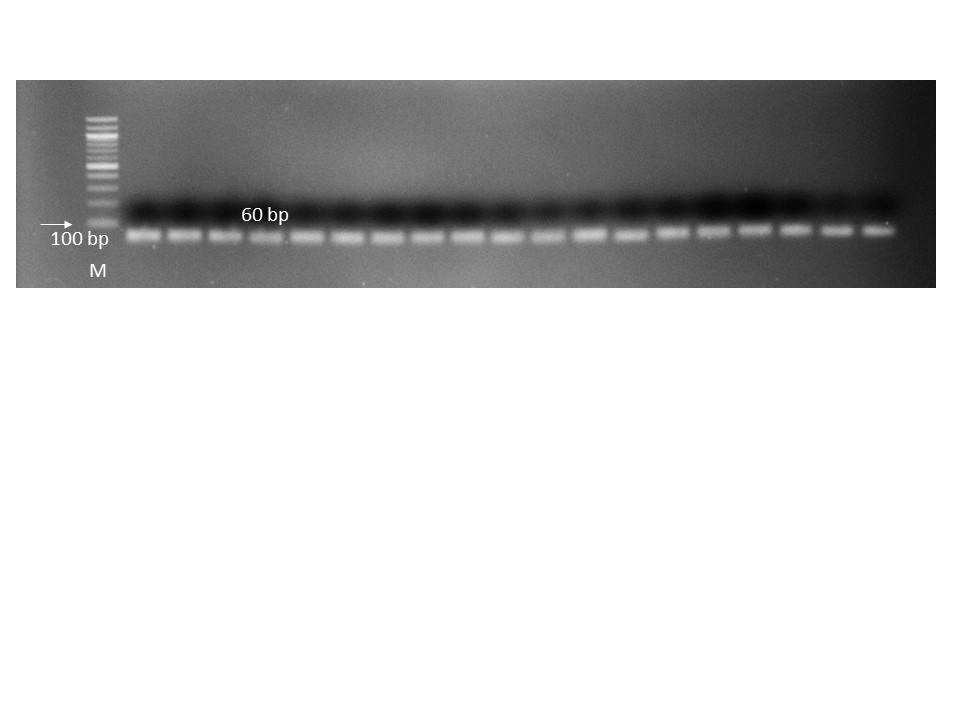


**Supplementary Figure S1 |** *GAPDH* amplicon with single PCR product of 60 bp indicating specific amplification. M: molecular marker.


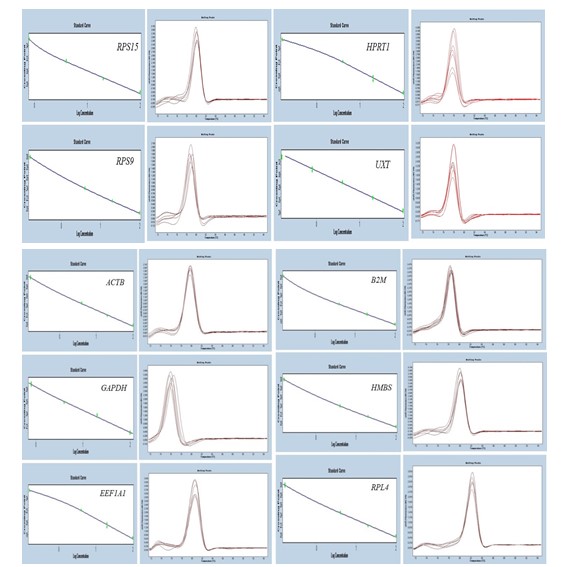
 **Supplementary Figure S2 |** qPCR standard curves and single melt curve peak for 10 reference genes.


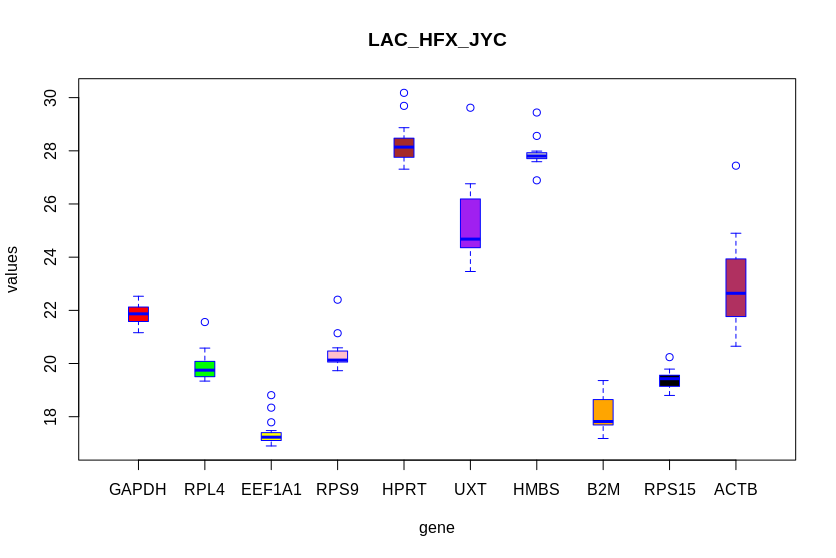


**Supplementary Figure S3** **|** Expression levels of individual candidate RGs in PBMCs of cold arid hypoxia group. The data are presented as quantification cycle (Cq) values of each gene in the box-whisker diagram. The median is shown as a line across the box while whiskers indicate maximum and minimum values.


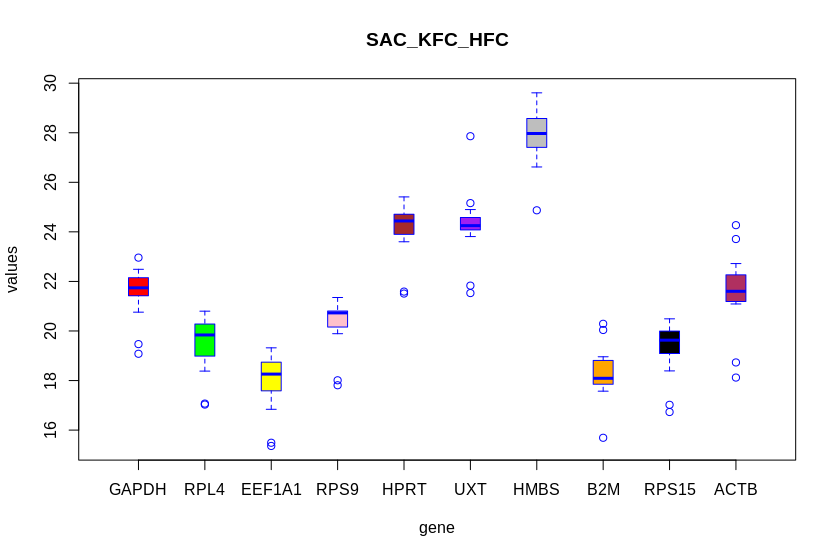


**Supplementary Figure S4 |** Expression levels of individual candidate RGs in PBMCs of hot arid normoxia group. The data are presented as quantification cycle (Cq) values of each gene in the box-whisker diagram. The median is shown as a line across the box while whiskers indicate maximum and minimum values.

**Supplementary Table S1 |** Average raw Ct values of individual RGs across all PBMCs in the cold arid hypoxia group

| **S.no** | **ANIMAL ID** | ***GAPDH*** | ***RPL4*** | ***EEF1A1*** | ***RPS9*** | ***HPRT*** | ***UXT*** | ***HMBS*** | ***B2M*** | ***RPS15*** | ***ACTB*** |
| --- | --- | --- | --- | --- | --- | --- | --- | --- | --- | --- | --- |
| 1 | LAC1 | 22.17 | 21.56 | 18.81 | 22.40 | 30.18 | 26.07 | 29.44 | 19.36 | 19.51 | 24.35 |
| 2 | LAC2 | 21.87 | 20.06 | 17.32 | 20.15 | 28.38 | 23.86 | 27.99 | 18.20 | 19.79 | 22.88 |
| 3 | LAC3 | 22.08 | 19.67 | 16.90 | 19.98 | 27.95 | 24.18 | 27.63 | 18.72 | 19.55 | 23.52 |
| 4 | LAC4 | 22.30 | 20.15 | 17.26 | 19.87 | 28.14 | 24.55 | 27.64 | 18.57 | 19.57 | 24.66 |
| 5 | LAC5 | 22.53 | 19.61 | 17.09 | 20.04 | 28.87 | 24.68 | 27.59 | 18.75 | 19.21 | 22.64 |
| 6 | LAC6 | 22.07 | 19.75 | 17.23 | 20.41 | 29.69 | 24.51 | 27.79 | 17.74 | 19.45 | 24.90 |
| 7 | HFX1 | 21.52 | 19.35 | 16.90 | 19.73 | 27.46 | 24.30 | 27.80 | 17.61 | 18.80 | 21.33 |
| 8 | HFX2 | 21.16 | 19.40 | 17.21 | 20.13 | 27.31 | 24.83 | 27.94 | 17.18 | 19.09 | 20.65 |
| 9 | HFX3 | 21.64 | 19.71 | 17.12 | 20.07 | 27.71 | 24.41 | 27.77 | 17.82 | 19.12 | 21.16 |
| 10 | HFX4 | 21.66 | 19.76 | 17.00 | 20.13 | 28.55 | 23.46 | 27.81 | 17.66 | 18.93 | 21.71 |
| 11 | HFX5 | 21.90 | 19.78 | 17.48 | 20.08 | 27.83 | 26.76 | 27.92 | 17.81 | 19.38 | 22.36 |
| 12 | JYC1 | 21.71 | 19.39 | 17.25 | 20.13 | 28.40 | 25.27 | 27.77 | 17.64 | 19.16 | 21.82 |
| 13 | JYC2 | 21.41 | 20.10 | 17.79 | 20.59 | 27.80 | 26.42 | 27.87 | 17.72 | 19.74 | 22.03 |
| 14 | JYC3 | 21.44 | 19.34 | 17.21 | 20.53 | 27.43 | 26.31 | 26.89 | 17.82 | 19.43 | 27.44 |
| 15 | JYC4 | 22.24 | 20.58 | 18.34 | 21.14 | 28.26 | 29.62 | 28.56 | 18.85 | 20.24 | 22.93 |
|  | **Average** | **21.84** | **19.88** | **17.39** | **20.36** | **28.26** | **25.28** | **27.89** | **18.09** | **19.40** | **22.96** |

**Supplementary Table S2 |** The average raw Ct values of individual RGs across all PBMCs in the hot arid normoxia group

| **S.no** | **ANIMAL ID** | ***GAPDH*** | ***RPL4*** | ***EEF1A1*** | ***RPS9*** | ***HPRT*** | ***UXT*** | ***HMBS*** | ***B2M*** | ***RPS15*** | ***ACTB*** |
| --- | --- | --- | --- | --- | --- | --- | --- | --- | --- | --- | --- |
| 1 | SAC1 | 21.60 | 20.38 | 18.34 | 20.40 | 24.43 | 27.86 | 28.21 | 18.09 | 19.83 | 21.60 |
| 2 | SAC2 | 21.87 | 20.18 | 18.36 | 20.63 | 24.95 | 23.99 | 27.97 | 18.04 | 19.39 | 21.09 |
| 3 | SAC3 | 22.96 | 20.04 | 18.70 | 20.73 | 24.09 | 24.36 | 28.57 | 18.91 | 19.72 | 23.71 |
| 4 | SAC4 | 22.49 | 20.65 | 18.78 | 20.84 | 24.64 | 24.63 | 28.68 | 18.29 | 20.11 | 22.49 |
| 5 | SAC5 | 22.23 | 20.80 | 19.10 | 21.00 | 24.91 | 24.90 | 28.90 | 18.71 | 20.49 | 22.04 |
| 6 | KFC1 | 22.07 | 19.84 | 18.18 | 20.73 | 24.58 | 24.25 | 27.81 | 18.10 | 19.88 | 21.79 |
| 7 | KFC2 | 21.63 | 19.73 | 19.32 | 20.77 | 24.44 | 24.17 | 27.51 | 17.75 | 19.63 | 21.18 |
| 8 | KFC3 | 21.45 | 19.24 | 17.66 | 19.89 | 23.60 | 24.23 | 27.31 | 17.73 | 18.97 | 21.20 |
| 9 | KFC4 | 21.39 | 18.73 | 17.51 | 19.92 | 23.71 | 23.81 | 26.99 | 17.57 | 18.39 | 21.41 |
| 10 | KFC5 | 21.74 | 19.65 | 17.94 | 20.65 | 24.40 | 24.30 | 28.04 | 17.95 | 19.54 | 21.51 |
| 11 | HFC1 | 19.47 | 17.07 | 15.49 | 18.01 | 21.51 | 21.83 | 26.62 | 15.69 | 17.02 | 18.73 |
| 12 | HFC2 | 22.44 | 20.56 | 19.27 | 21.35 | 25.41 | 25.16 | 28.58 | 20.29 | 20.32 | 22.72 |
| 13 | HFC3 | 19.08 | 17.03 | 15.36 | 17.81 | 21.59 | 21.53 | 24.87 | 17.95 | 16.73 | 18.12 |
| 14 | HFC4 | 20.76 | 18.38 | 16.84 | 21.11 | 24.78 | 24.53 | 29.61 | 20.04 | 20.43 | 24.27 |
| 15 | HFC5 | 21.95 | 19.90 | 18.26 | 20.73 | 24.61 | 24.25 | 27.72 | 18.96 | 19.21 | 21.78 |
|  | **Average** | **21.54** | **19.48** | **17.94** | **20.30** | **24.11** | **24.25** | **27.82** | **18.27** | **19.31** | **21.57** |

**Supplementary Table S3 |** Relative expression, standard deviation (SD), and standard error (SE) of target genes in high-altitude and low-altitude cattle populations

|  | High-altitude (HA) cattle | | | Low-altitude (LA) cattle | | |
| --- | --- | --- | --- | --- | --- | --- |
| Target  genes | Relative  expression | SD | SE | Relative  expression | SD | SE |
| *HIF1A* | 4.470 | 0.837 | 0.216 | 1.162 | 0.551 | 0.142 |
| *EPAS1* | 4.879 | 0.931 | 0.240 | 1.425 | 0.912 | 0.236 |
| *HSP70* | 0.789 | 0.307 | 0.079 | 1.135 | 0.453 | 0.117 |
| *HSP27* | 0.448 | 0.213 | 0.055 | 1.178 | 0.702 | 0.181 |

**Supplementary Table S4 |** Relative expression, standard deviation (SD), and standard error (SE) of target genes post normalization with least stable reference genes

|  | High altitude (HA) | | | Low altitude (LA) | | |
| --- | --- | --- | --- | --- | --- | --- |
| Target  genes | Relative  expression | SD | SE | Relative  expression | SD | SE |
| *HIF1A* | 30.745 | 20.782 | 5.366 | 1.317 | 0.952 | 0.246 |
| *EPAS1* | 40.560 | 15.726 | 4.060 | 1.425 | 0.875 | 0.226 |
| *HSP70* | 6.624 | 8.310 | 2.146 | 1.183 | 0.611 | 0.158 |
| *HSP27* | 2.972 | 2.575 | 0.665 | 1.174 | 0.667 | 0.172 |
